# Anisotropic to Isotropic Transition in Monolayer Group-IV Tellurides

**DOI:** 10.3390/ma14164495

**Published:** 2021-08-11

**Authors:** Qian Wang, Liyuan Wu, Alexander Urban, Huawei Cao, Pengfei Lu

**Affiliations:** 1 State Key Laboratory of Information Photonics and Optical Communications, Beijing University of Posts and Telecommunications, Beijing 100876, China; wq940411@bupt.edu.cn; 2 CAS Key Laboratory for Biomedical Effects of Nanomaterials and Nanosafety, Institute of High Energy Physics, Chinese Academy of Sciences, Beijing 100049, China; wuly2018@gmail.com; 3 Department of Chemical Engineering, Columbia University, New York, NY 10027, USA; au2229@columbia.edu; 4 State Key Laboratory of Computer Architecture, Institute of Computing Technology, Chinese Academy of Sciences, Beijing 100190, China

**Keywords:** two-dimensional materials, group-IV tellurides, anisotropic structure

## Abstract

Monolayer group-IV tellurides with phosphorene-derived structures are attracting increasing research interest because of their unique properties. Here, we systematically studied the quasiparticle electronic and optical properties of two-dimensional group-IV tellurides (SiTe, GeTe, SnTe, PbTe) using the GW and Bethe–Salpeter equation method. The calculations revealed that all group-IV tellurides are indirect bandgap semiconductors except for monolayer PbTe with a direct gap of 1.742 eV, while all of them are predicted to have prominent carrier transport ability. We further found that the excitonic effect has a significant impact on the optical properties for monolayer group-IV tellurides, and the predicted exciton binding energy is up to 0.598 eV for SiTe. Interestingly, the physical properties of monolayer group-IV tellurides were subject to an increasingly isotropic trend: from SiTe to PbTe, the differences of the calculated quasiparticle band gap, optical gap, and further exciton binding energy along different directions tended to decrease. We demonstrated that these anisotropic electronic and optical properties originate from the structural anisotropy, which in turn is the result of Coulomb repulsion between non-bonding electron pairs. Our theoretical results provide a deeper understanding of the anisotropic properties of group-IV telluride monolayers.

## 1. Introduction

Group IV–VI compounds, such as PbTe, GeTe, and SnTe, have been used as the functional materials in optoelectronic and thermoelectric devices for a long time because of their effective suitable bandgaps and high Seebeck coefficients [1,2,3]. Following the discovery of graphene, in recent years there has been tremendous interest in two-dimensional materials, owing to their unusual electronic and optical properties with potential applications in solar cells, field-effect transistors, and catalysts [4,5,6,7,8,9,10]. In particular, group IV–VI semiconductors with layered structures and weak van der Waals interlayer interactions allowing for isolation by mechanical exfoliation have been considered as free-standing monolayers [11,12,13,14,15,16,17]. In addition, various two-dimensional nanosheets have already been successfully synthesized using chemical vapor deposition, liquid phase exfoliation, and spark plasma sintering techniques, rendering them potentially useful candidates for large-scale manufacturing and device applications [18,19,20,21,22,23,24,25,26,27].

Two-dimensional IV–VI compounds exhibit structural polymorphism, whereas almost all of the bulk compounds form in a distorted NaCl type structure, i.e., a black phosphorene-derived structure, which is consistent with the NaCl or distorted NaCl structure in their bulk case [13,28,29]. In group-IV monochalcogenide monolayers, atoms of the different species occupy the two inequivalent phosphorus sites in the phosphorene structure, respectively. The anisotropic structure of phosphorene gives rise to a distinct anisotropy in conductance, effective carrier masses, and optical response [30,31,32,33,34]. As isoelectronic compounds of phosphorene, two-dimensional group-IV monochalcogenides with such distorted NaCl type structures are expected to possess interesting anisotropic properties. For example, for applications such as thermoelectric materials, the anisotropic phosphorene structure of the group-IV monochalcogenides brings about the anisotropy of the transport coefficient [35,36]. Furthermore, the structure also results in an anisotropic response to in-plane strains in the electronic and optical properties of group-IV monochalcogenides monolayers [37,38]. Yang et al. reported few-layer SnSe-based field-effect transistors with a high anisotropic ratio of carrier mobility between different directions [39]. Recently, the anisotropic plasmon response of two-dimensional GeSe nanoribbon has been demonstrated, which provides promising application potential in novel polarization-dependent optoelectronic devices [40]. The structural anisotropy could also play an important role in determining the ferroelectricity of monolayer group-IV tellurides. In fact, monolayers GeS, GeSe, SnS, and SnSe have been theoretically predicted to undergo large in-plane spontaneous polarization, which usually indicates the emergence of ferroelectricity [41,42,43]. Chang et al. experimentally reported the discovery of robust ferroelectricity in atomic-thick SnTe, down to a one-unit cell limit [21]. Zhang et al. predicted that the unequal lattice constants and relative atomic displacements are responsible for ferroelectricity in monolayer GeTe, SnTe, and the non-ferroelectric nature of monolayer PbTe [44]. These findings indicate that structural anisotropy has a profound influence on the physical properties of this system.

A number of theoretical studies of the physical properties of some two-dimensional group-IV tellurides have been published [45,46,47,48]; however, a systematic investigation of the effect of the anisotropic structure on the electronic and optical properties of monolayer group-IV tellurides is still lacking. In this paper, we systematically study the quasiparticle band structures and optical properties of group-IV tellurides (SiTe, GeTe, SnTe, PbTe) by means of predictive calculations based on the accurate many-body perturbation GW theory and the Bethe–Salpeter equation. We find that all group-IV tellurides display anisotropic electronic and optical properties, except monolayer PbTe. From monolayer SiTe to PbTe, there is an increasingly isotropic tendency in the quasiparticle electronic and optical properties, which is consistent with the change of the crystal structure. To obtain insights into the structural anisotropy, we explore the bond nature in the compounds by analyzing the electron localization functions and charge density.

## 2. Computational Details

Density functional theory (DFT) [49,50] calculations were performed by adopting the generalized gradient approximation (GGA) of the Perdew–Burke–Ernzerhof (PBE) functional [51] for the exchange-correlation energy as implemented in the Vienna ab initio Simulation Package (VASP) [52,53]. Projector augmented-wave (PAW) pseudopotentials [54] were used with a plane-wave basis set with an energy cutoff of 500 eV. The first Brillouin zone was sampled with a 12 × 10 × 1 Monkhorst–Pack mesh [55] for the calculation of group-IV tellurides. A vacuum region larger than 15 Å was used to eliminate the interaction between the adjacent periodic images of the monolayers. All unit cells were fully relaxed until the forces were well converged below 0.001 eV/Å and the total energy changes were less than 10^−5^ eV. To overcome the problem of bandgap underestimation in DFT–GGA calculations, we employed the G_0_W_0_ approach [56,57] with initial PBE wavefunctions to calculate the quasiparticle (QP) electronic structures. Starting from the G_0_W_0_ calculations, the QP band structures were interpolated using the maximally localized Wannier functions (MLWFs) approach [58,59] implemented in the Wannier90 package [60], and the *s* and *p* orbitals of group-IV and Te atoms were chosen for the initial projections. To calculate accurate optical band gaps, the electron-hole Coulomb interactions were taken into account by solving the Bethe–Salpeter equation (BSE) [56,57]. The random-phase approximation (RPA) was also employed in the optical property calculations for comparison [61,62]. The charge transfer between group-IV and Te atoms was calculated according to the Bader charge method [63]. The crystal structures, electron localization functions, and electronic charge densities of these group-IV telluride monolayers were plotted using the VESTA visualization program [64].

## 3. Results and Discussion

### 3.1. Geometry Structures

**Figure 1:**
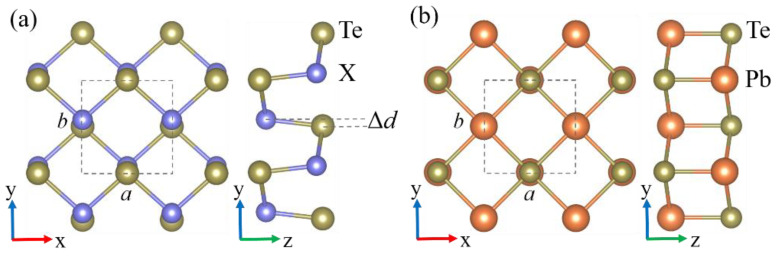
Top and side views of the monolayer (**a**) XTe (X = Si, Ge, Sn) and (**b**) PbTe structures. Δ*d* is the relative displacement between the group-IV and Te atoms along the y direction. The unit cell is indicated by a dashed rectangle.

First, we considered the structural properties of group-IV telluride monolayers. Previous research has established that group-IV telluride monolayers are dynamically stable in principle with the NaCl or distorted NaCl atomic arrangement [44,45,65,66]. In this system, the crystal structure of XTe (X = Si, Ge, Sn) belongs to the Pmn2_1_ space group with a relative displacement Δ*d* between the group-IV atom and the Te atom along the y direction, while two-dimensional PbTe forms in a different P4/nmm structure without relative displacement [44], i.e., as a cleaved monolayer with a cubic NaCl structure shown in Figure 1. The optimized structural parameters for SiTe (*a* = 4.11 Å, *b* = 4.29 Å), GeTe (*a* = 4.23 Å, *b* = 4.39 Å), SnTe (*a* = 4.55 Å, *b* = 4.59 Å) and PbTe (*a* = *b* = 4.64 Å) are summarized in Table 1, which were in good agreement with previous theoretical reports [29,67,68]. Note that the lattice constants increased gradually from monolayer SiTe to PbTe. However, the relative atomic displacements Δ*d* showed a decreasing trend from 0.31 Å to 0.00 Å as the atomic number of the group-IV species increased, so that the monolayer PbTe (with Δ*d* = 0.00 Å) formed in an ideal NaCl-type structure. Its isotropic structure gives rise to the non-ferroelectric character of monolayer PbTe that distinguishes it from other monolayer group-IV tellurides, which has been reported in previous theoretical work [21,44]. Note that the lattice constant ratio (*b/a*) followed a similar trend as Δ*d*, and thus the two-dimensional group-IV telluride configurations tended to become more isotropic descending the group from SiTe to PbTe.

### 3.2. Quasiparticle Band Structures

**Figure 2:**
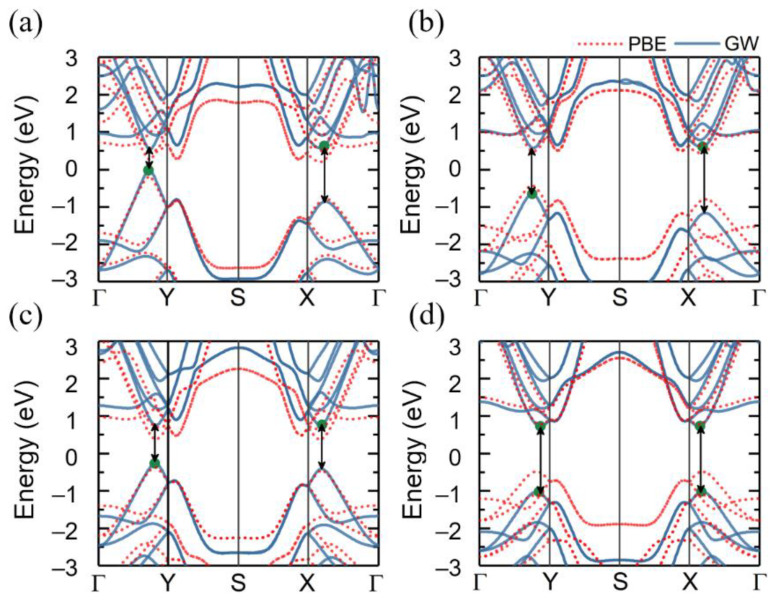
Calculated band structures as obtained from the PBE functional (red dotted lines) and from the GW method (blue lines) for monolayers (**a**) SiTe (**b**) GeTe (**c**) SnTe (**d**) PbTe. Green circles indicate the global CBM or VBM. Local direct transitions are indicated by arrows.

Next, we investigated the quasiparticle band structures of the monolayer group-IV tellurides. The electronic band structures of the monolayer group-IV tellurides as obtained from both PBE calculations and from the one-shot G_0_W_0_ approximation are shown in Figure 2. It was found that the shape of the G_0_W_0_ bands closely followed the PBE results with an increase in the bandgap. All group-IV tellurides, except for PbTe, possessed an indirect bandgap with the conduction band minimum (CBM) localized along the Γ-X line of the Brillouin zone and the valence band maximum (VBM) at a point along Γ-Y. Monolayer PbTe exhibited a direct bandgap with a PBE value of 1.26 eV along the Γ-X (Y) direction, which is consistent with previous theoretical results [13].

Within the PBE method, the minimum indirect band gaps for the monolayers SiTe, GeTe, and SnTe were 0.40 eV, 0.87 eV, and 0.74 eV, and thus did not follow a monotonous trend. This anomalous behavior is different from the trends seen for the band gaps of the transition metal dichalcogenides and group-III chalcogenides and has previously been attributed to the balance between the relative atomic energy levels and the repulsion between the levels [45,69,70].

XTe (X = Si, Ge, Sn) exhibited a similar feature in their band structures in which local CBM (VBM) were located along the Γ-X direction and the Γ-Y direction. The calculated quasiparticle gaps along both the x and y directions are indicated in Figure 2 and summarized in Table 2. The band gaps were found to be anisotropic with the E_g_(x) > E_g_(y), and the differences in the gaps between the different directions for the monolayers SiTe, GeTe, SnTe, and PbTe were 0.876 eV, 0.528 eV, 0.139 eV, and 0.000 eV, respectively. In addition, we also considered the spin–orbit coupling (SOC) effect on the electronic properties of monolayer group-IV tellurides. The comparison of the quasiparticle band structures with and without including the SOC is shown in Figure S1. It can be seen that the SOC effect had a very limited impact on the band structures except for the PbTe monolayer. After turning on SOC, the PbTe monolayer presented a reduced GW band gap of 1.23 eV. According to the previous reports, the reduced band gap with the SOC effect in the PbTe monolayer is relevant to the band inversion around the band edge [71].

We next estimated the hole (*m_h_*) and electron effective mass (*m_e_*) along the x and y directions according to the equation $\frac{1}{m}=\frac{1}{\hbar^{2}}\cdot \frac{d^{2}E}{dk^{2}}$, and the results are also listed in Table 2. The effective masses of the group-IV telluride monolayers were in the range of 0.09~0.24 *m*_0_ for electrons and 0.09~0.28 *m*_0_ for holes, respectively, which are very close to the previous reports [55]. In particular, monolayer SnTe had a low effective mass of 0.11 *m*_0_ (0.10 *m*_0_) and 0.13 *m*_0_ (0.11 *m*_0_) for electrons and holes along the x (y) direction, indicating its excellent carrier transportability and possible application in novel electronic devices. The calculated effective masses of the charge carriers along the x direction were higher than those in the y direction for all considered structures, and the difference between different directions tended to decrease as the atomic number of the group-IV species increased.

### 3.3. Optical Properties of Monolayer Group-IV Tellurides

In addition to the electronic properties, we also considered the optical properties of the monolayer group-IV tellurides. Owing to the reduced dimensionality and depressed screening in monolayer materials, excitonic effects can substantially reshape the optical spectra [72,73].

**Figure 3:**
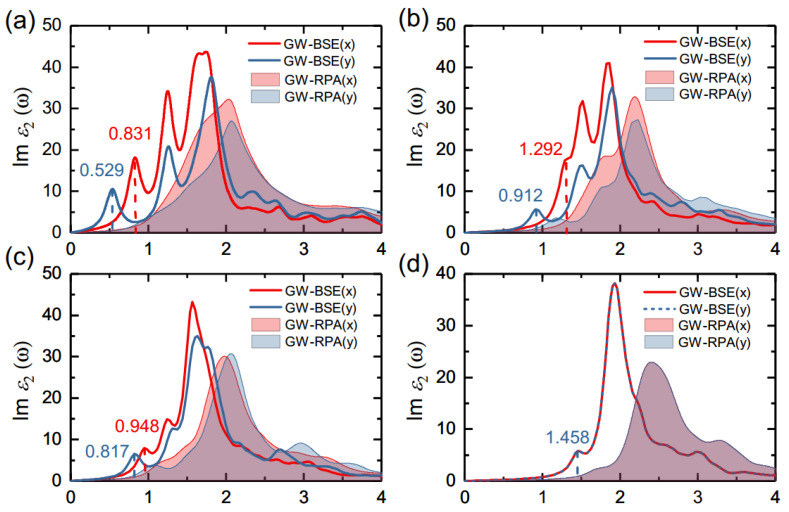
Imaginary part of the dielectric functions of monolayers (**a**) SiTe, (**b**) GeTe, (**c**) SnTe, and (**d**) PbTe with (GW–BSE) and without (GW–RPA) e–h interactions for linearly polarized light along the x and y directions.

In order to obtain accurate absorption spectra, we took the electron–hole (e–h) Coulomb interactions into account by using the GW–BSE approach. For comparison, we also evaluated absorption spectra without e–h interactions using the RPA approach. The absorption spectra of the monolayer group-IV tellurides with (labeled as “GW–BSE”) and without (labeled as “GW–RPA”) e–h interactions are shown in Figure 3. As seen in Figure 3, all spectra exhibited red-shift when the e–h interactions were considered, indicating that the excitonic effects significantly modified the optical properties of the monolayers.

Within the BSE approach, we noted that there were two distinct absorption peaks before the maximum intensity peak, except for the PbTe monolayer. Here we took the monolayer SiTe for an example. In Figure 3a, it can be seen that the first optical excitation occurred at 0.83 eV for the x direction and at 0.53 eV for the y direction, and the optical spectra of monolayer SiTe exhibited obvious anisotropic behavior. The first significant absorption peaks corresponded to the minimum direct transitions of 1.43 eV along the Γ-X direction and of 0.56 eV along the Γ-Y direction, respectively, as indicated by the black arrows in Figure 2a.

The exciton binding energy, which describes the strength of excitonic effects, can be calculated from the energy difference between the GW–BSE optical absorption energy and the direct quasiparticle band gap. The calculated exciton binding energies for the lowest exciton states of monolayer SiTe along the x and y directions were 0.60 eV and 0.03 eV, respectively. This result reveals a strongly bound exciton in the x direction, but not in the y direction.

Similarly, the calculated exciton binding energies along the x and y directions were 0.47, 0.31 eV for monolayer GeTe, and 0.22, 0.21 eV for monolayer SnTe, as reported in Table 2, which are in reasonable agreement with previous theoretical results (0.34, 0.23 eV for GeTe, and 0.19, 0.19 eV for SnTe) [67]. However, for PbTe, which has an isotropic structure, the optical spectrum in the x direction coincided exactly with that in the y direction, and the exciton binding energy was estimated to be 0.28 eV. The considered monolayer group-IV tellurides had exciton binding energies ranging from 0.2 to 0.6 eV, implying a promising potential for optoelectronic applications.

### 3.4. Anisotropic to Isotropic Transition

As discussed above, the crystal structures of the group-IV telluride monolayers show an anisotropic-to-isotropic transition from SiTe to PbTe, and the differences in the various band gaps and carrier effective masses tended to decrease, indicating a more isotropic behavior. Similarly, the optical properties of monolayer group-IV tellurides experienced a similar change from anisotropic to isotropic with an increase in the atomic number, as seen in Figure 3.

In order to identify the relationship between the more isotropic physical properties and the crystal structures, we replaced Si with Pb in the anisotropic monolayer SiTe crystal structure and recalculated the physical properties (Figures S2 and S3). Within the GW approximation, the minimum direct band gaps along the x and y directions were 1.11 eV and 1.57 eV, respectively, and the absorption spectra in different directions did not coincide. Hence, the physical properties experienced an obvious anisotropy in this modified PbTe structure, and we can thus conclude that the anisotropy of the electronic and optical properties in the monolayer group-IV tellurides is a consequence of the structural anisotropy.

The question remains as to what causes the anisotropic to the isotropic transition of the crystal structures. To better understand the trends in the crystal structures, we investigated the atomic bonding characteristics by evaluating the electron localization functions (ELF) [74]. The ELF is a dimensionless localization index restricted to the range [0,1] that indicates the extent of spatial localization of the electrons by measuring the same-spin probability relative to the uniform-density electron gas. A value of 0 denotes the absence of electrons, 1 corresponds to complete localization, and 0.5 indicates the uniform free electron gas. Considering a similar crystal structure, we also calculated the ELF of black phosphorene for comparison. 

**Figure 4:**
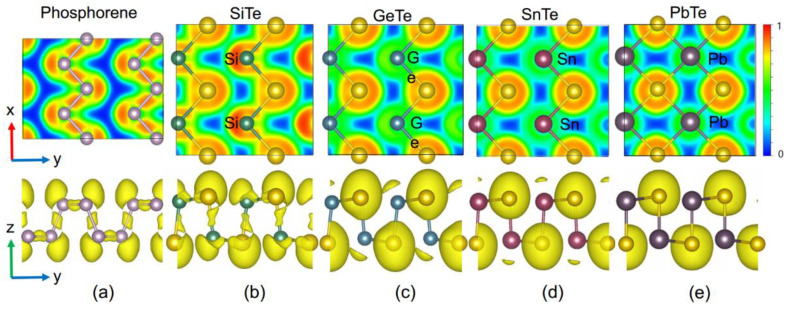
ELF maps sliced across the Te layers (top panel) and side views of the ELF with isosurface value 0.64 (bottom panel) for the monolayer (**a**) SiTe, (**b**) GeTe, (**c**) SnTe, and (**d**) PbTe. Increasing electron localization from 0 to 1 is plotted with colors from blue to red.

The ELF of the four two-dimensional group-IV telluride configurations and of black phosphorene is shown in Figure 4. As seen in the figure, monolayer SiTe exhibited similar bonding features as phosphorene in the following two aspects: (1) High values of ELF existing along lines connecting pairs of atoms, implying the formation of covalent Si–Te bonds. (2) The Coulomb repulsion among non-bonding electron pairs surrounding the Si and Te atoms leading to a relative atomic displacement and causing the anisotropic structure. For monolayer GeTe and SnTe, the characteristic of the covalent bond was not as obvious as for the SiTe monolayer with fewer non-bonding electrons surrounding the atoms.

In contrast, for the PbTe monolayer, there were no local maxima in the ELF along lines connecting pairs of atoms, and the Te atoms were surrounded by a nearly spherical density distribution with only a slight deformation along the direction to the surrounding group-IV atoms. This means the bonding in monolayer PbTe is significantly less covalent and rather more ionic in nature compared to SiTe and the other group-IV tellurides.

Additionally, compared to phosphorene, there was an electron localization tendency around the Te atoms in the group-IV tellurides, indicating an increased electron transfer from the group-IV atoms to the Te atoms, which is consistent with the calculated Bader charge transfer in Table 2. The more electrons localized around the Te atoms, instead of between the Te and group-IV atoms, the more ionic the bonding becomes. In PbTe, i.e., the structure dominated by ionic bonds, no significant Coulomb repulsion from non-bonding electron pairs was observed, and as a consequence, the structure showed no anisotropy, leading to isotropic electronic properties.

**Figure 5:**
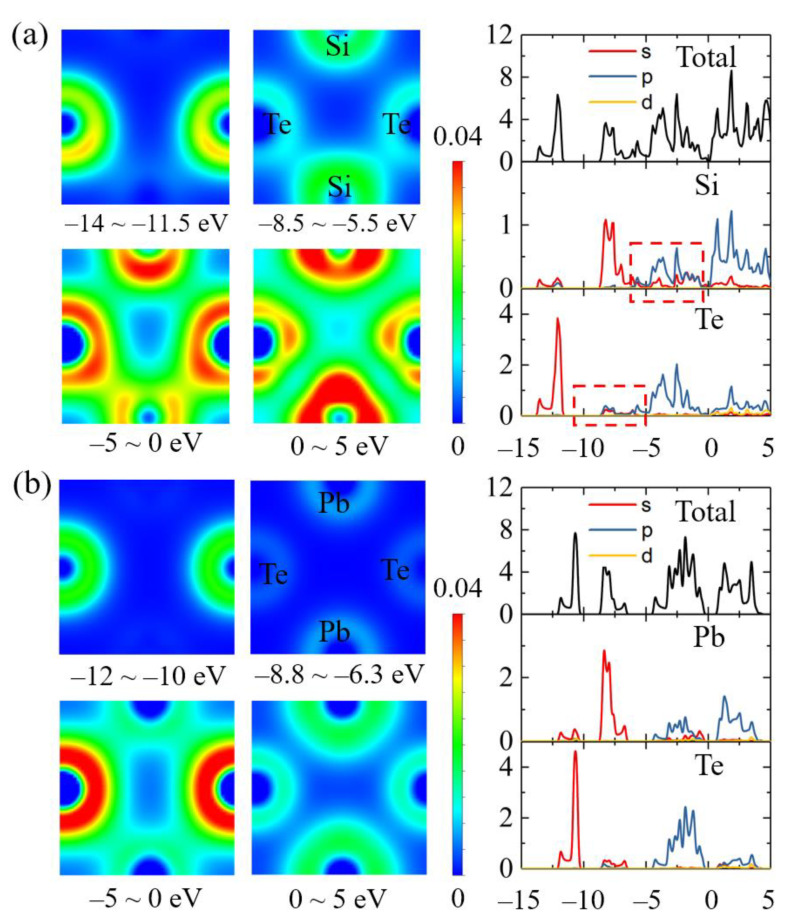
Electronic charge density for different energy ranges of the valence bands and conduction bands, and total and partial density of states for monolayer (**a**) SiTe and (**b**) PbTe.

To further improve the understanding of the difference in the bond nature among the group-IV tellurides, we plotted the projected density of states (PDOS) of monolayer SiTe and PbTe, and the corresponding charge density from different energy ranges as shown in Figure 5. The two compounds showed some similarities in the PDOS. It can be seen that the lowest regions of the valence band were mostly composed of Te 5s electronic states. The second-lowest states of the valence band had a major contribution from the group-IV atom’s s states with a little admixture of Te s and p states.

The difference in bonding character is apparent at the top of the valence bands (−5~0 eV). For the monolayer SiTe, the electronic charge density exhibited a more covalent bonding character, corresponding to the hybridization between Si s and p states in this region. However, the bottom region of the conduction bands (0~5 eV) corresponded to the ionicity of the monolayer group-IV tellurides by p–p ionic bonding. For the PbTe monolayer, the electronic charge density at the top of the valence bands showed no obvious charge sharing phenomena between Pb and Te atoms when compared with the SiTe monolayer; conversely, the p–p ionic bonding played a dominant role in this compound. Therefore, covalence and ionicity coexist in all the group-IV telluride monolayers with a black phosphorene-derived structure. For the SiTe monolayer, the s–p covalent bonding played a more important role than ionic bonding, while for the PbTe monolayer, the contribution from p–p ionic bonding was greater; thus, the compounds became less covalent and more ionic from SiTe to PbTe.

## 4. Conclusions

In summary, the quasiparticle electronic and optical properties of the monolayer group-IV tellurides with black-phosphorene derived structures were investigated using accurate first-principles calculations in the quasiparticle GW and Bethe–Salpeter equation (BSE) formalisms. Our calculations showed that there is an anisotropic to isotropic transition of the physical properties of group-IV telluride monolayers. The differences in the calculated quasiparticle bandgap, BSE optical gap, and exciton binding energy in different directions tended to decrease from SiTe to SnTe, while the band structure and optical property exhibited totally isotropic behaviors for PbTe monolayer, which is the consequence of vanishing structural anisotropy. By analyzing the electron localization function and charge density, we identified the change in the bonding behavior as the origin of this increasing isotropic trend. While covalent bonding dominated in SiTe, the bonding in the monolayer group-IV tellurides became more ionic as the atomic number increased, and the Coulomb repulsion between non-bonding electron pairs grew weaker. As a result, the crystal structures and the quasiparticle electronic and optical properties became more isotropic. This understanding clarifies the origin of the anisotropy in group-IV telluride monolayers and can guide the design of derived materials for applications in electronic and optical devices.

## Figures and Tables

**Table 1:** Structural parameters of phosphorene and group-IV telluride monolayers: lattice constants (*a*, *b*), lattice constant ratios (*b*/*a*), and relative displacements (Δ*d*) between group-IV and Te atoms along the *y* direction are given.

	*a* (Å)	*b* (Å)	*b*/*a*	Δ*d* (Å)
Phosphorene	3.30	4.63	1.403	0.83
SiTe	4.11	4.29	1.044	0.31
GeTe	4.23	4.39	1.038	0.28
SnTe	4.55	4.59	1.009	0.15
PbTe	4.64	4.64	1	0

**Table 2:** Calculated carrier effective masses (*m*), PBE gaps (*E_g_*^PBE^), GW gaps (*E_g_*^GW^), BSE optical gaps (*E_g_*^opt^), exciton binding energies (*E_b_*) and Bader charge transfer (Δ***ρ***) of monolayers SiTe, GeTe, SnTe, PbTe along different directions compared to previous reports.

	*m_e_* (*m*_0_)	*m_h_* (*m*_0_)	*E_g_*^PBE^ (eV)	*E_g_*^GW^ (eV)	*E_g_*^opt^ (eV)	*E_b_* (eV)	Δ*ρ* (e^−^)	Ref
Direction	*x*/*y*	*x*/*y*	*x*/*y*	*x*/*y*	*x*/*y*	*x*/*y*	-	
SiTe	0.24/0.09	0.21/0.09	0.99/0.47	1.43/0.56	0.83/0.53	0.60/0.03	0.37	
GeTe	0.24/0.14	0.28/0.13	1.24/0.92	1.76/1.24	1.29/0.91	0.47/0.31	0.39	
	-	-	-	1.68/1.23	1.34/1.00	0.34/0.23	-	ref [67]
SnTe	0.11/0.10	0.13/0.11	0.84/0.75	1.16/1.04	0.95/0.82	0.22/0.21	0.63	
	-	-	-	1.04/1.02	0.85/0.83	0.19/0.19	-	ref [67]
PbTe	0.20	0.18	1.26	1.74	1.46	0.28	0.66	
	-	-	1.3	-	-	-	-	ref [71]
	0.169	0.190	1.26	-	-	-	-	ref [13]

## Data Availability

The data presented in this study are available in the article and Supplementary Materials.

## Supplementary Materials

The following are available online at https://www.mdpi.com/article/10.3390/ma14164495/s1, Figure S1: Calculated band structures as obtained from the GW method with (blue lines) and without spin-orbit coupling (red dotted lines) for monolayer (**a**) SiTe (**b**) GeTe (**c**) SnTe (**d**) PbTe, Figure S2: Calculated band structure as obtained from the PBE functional (red dotted lines) and from the GW method (blue lines) for the modified PbTe structure, Figure S3: Imaginary part of the dielectric functions of the modified PbTe structure with (GW-BSE) and without (GW-RPA) electron-hole interaction for linearly polarized light along the x and y directions.
