# Supplementary material for: Anisotropic to Isotropic Transition in Monolayer Group-IV Tellurides
Source: Materials (Basel). 2021 Aug 11;14(16):4495. doi: 10.3390/ma14164495 (PMC8398135; doi:10.3390/ma14164495)
Supplement: Supplementary file 1 [file materials-14-04495-s001.zip › materials-1285048-supplementary.pdf]

materials

Supplementary Materials

Anisotropic to Isotropic Transition in Monolayer Group-IV Tellurides

Qian Wang [1], Liyuan Wu [2], Alexander Urban [3], Huawei Cao [4,*], Pengfei Lu [1,*]

1 State Key Laboratory of Information Photonics and Optical Communications, Beijing University of Posts and Telecommunications, Beijing 100876, China; wq940411@bupt.edu.cn
2 CAS Key Laboratory for Biomedical Effects of Nanomaterials and Nanosafety, Institute of High Energy Physics, Chinese Academy of Sciences, Beijing 100049, China; wuly2018@gmail.com
3 Department of Chemical Engineering, Columbia University, New York, NY 10027, USA; au2229@columbia.edu
4 State Key Laboratory of Computer Architecture, Institute of Computing Technology, Chinese Academy of Sciences, Beijing 100190, China
* Correspondence: caohuawei@ict.ac.cn (H.C.); photon.bupt@gmail.com (P.L.)

Citation: Wang, Q.; Wu, L.; Urban, A.; Cao, H.; Lu, P. Anisotropic to Isotropic Transition in Monolayer Group-IV Tellurides. *Materials* **2021**, *14*, 4495. https://doi.org/10.3390/ma14164495

Academic Editor: Albena Paskaleva

Received: 18 June 2021
Accepted: 6 August 2021
Published: 11 August 2021

Publisher's Note: MDPI stays neutral with regard to jurisdictional claims in published maps and institutional affiliations.

Copyright: © 2021 by the authors. Submitted for possible open access publication under the terms and conditions of the Creative Commons Attribution (CC BY) license (http://creativecommons.org/licenses/by/4.0/).

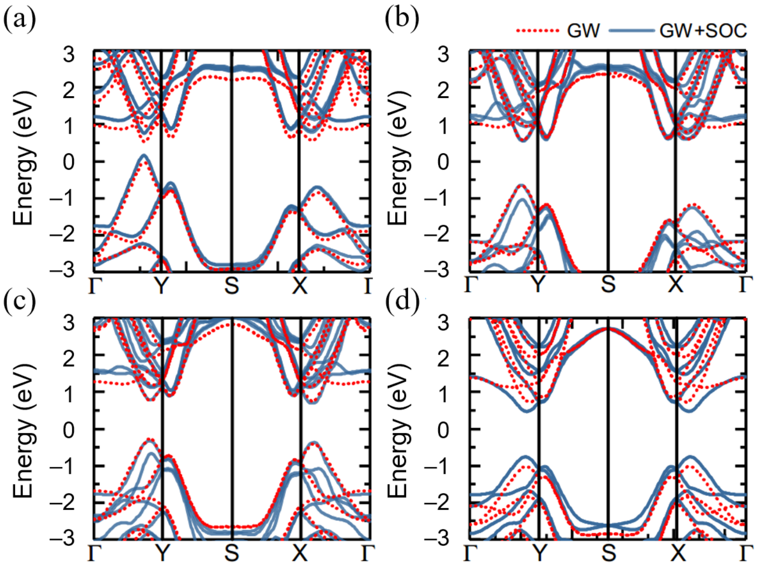

Figure S1. Calculated band structures as obtained from the GW method with (blue lines) and without spin-orbit coupling (red dotted lines) for monolayer (**a**) SiTe (**b**) GeTe (**c**) SnTe (**d**) PbTe.

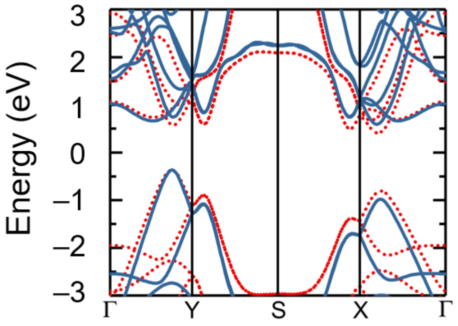

Figure S2. Calculated band structure as obtained from the PBE functional (red dotted lines) and from the GW method (blue lines) for the modified PbTe structure.

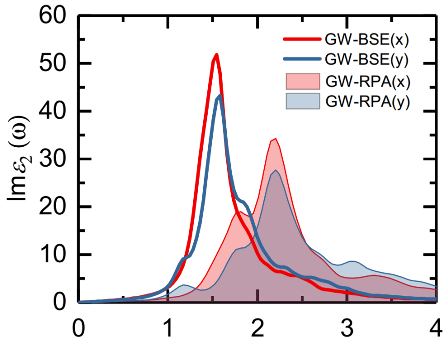

Figure S3. Imaginary part of the dielectric functions of the modified PbTe structure with (GW-BSE) and without (GW-RPA) electron-hole interactions for linearly polarized light along the *x* and *y* directions.
